# Characterization of Glutathione Peroxidase 4 in Rat Oocytes, Preimplantation Embryos, and Selected Maternal Tissues during Early Development and Implantation

**DOI:** 10.3390/ijms22105174

**Published:** 2021-05-13

**Authors:** Andrea Kreheľová, Veronika Kovaříková, Iveta Domoráková, Peter Solár, Alena Pastornická, Andriana Pavliuk-Karachevtseva, Silvia Rybárová, Ingrid Hodorová, Jozef Mihalik

**Affiliations:** 1 Department of Anatomy, Medical Faculty, Šafárik University, Šrobárova 2, 041 83 Košice, Slovakia; andrea.krehelova@student.upjs.sk (A.K.); alena.pastornicka@student.upjs.sk (A.P.); andriana.pavliuk-karachevtseva@upjs.sk (A.P.-K.); silvia.rybarova@upjs.sk (S.R.); ingrid.hodorova@upjs.sk (I.H.); 2 Institute of Animal Physiology, Centre of Biosciences, Slovak Academy of Sciences, Šoltésovej 4-6, 040 01 Košice, Slovakia; kovarikova@saske.sk; 3 Department of Histology and Embryology, Medical Faculty, Šafárik University, Šrobárova 2, 041 83 Košice, Slovakia; iveta.domorakova@upjs.sk; 4 Department of Medical Biology, Medical Faculty, Šafárik University, tr. SNP 1, 040 11 Košice, Slovakia; peter.solar@upjs.sk

**Keywords:** antioxidant, glutathione peroxidase, infertility, oxidative stress, reactive oxygen species, reproductive system

## Abstract

This study aimed to describe glutathione peroxidase 4 (GPx4) in rat oocytes, preimplantation embryos, and female genital organs. After copulation, Sprague Dawley female rats were euthanized with anesthetic on the first (D1), third (D3), and fifth days of pregnancy (D5). Ovaries, oviducts, and uterine horns were removed, and oocytes and preimplantation embryos were obtained. Immunohistochemical, immunofluorescent, and Western blot methods were employed. Using immunofluorescence, we detected GPx4 in both the oocytes and preimplantation embryos. Whereas in the oocytes, GPx4 was homogeneously diffused, in the blastomeres, granules were formed, and in the blastocysts, even clusters were present mainly around the cell nuclei. Employing immunohistochemistry, we detected GPx4 inside the ovary in the corpus luteum, stroma, follicles, and blood vessels. In the oviduct, the enzyme was present in the epithelium, stroma, blood vessels, and smooth muscles. In the uterus, GPx4 was found in the endometrium, myometrium, blood vessels, and stroma. Moreover, we observed GPx4 positive granules in the uterine gland epithelium on D1 and D3 and cytoplasm of fibroblasts forming in the decidua on D5. Western blot showed the highest GPx4 levels in the uterus and the lowest levels in the ovary. Our results show that the GPx4 is necessary as early as in the preimplantation development of a new individual because we detected it in an unfertilized oocyte in a blastocyst and not only after implantation, as was previously thought.

## 1. Introduction

Approximately 10% of the human population is infertile [1]. Couple infertility is defined as the inability to conceive even after one year of unprotected sexual intercourse [2].

In men, infertility is caused by various etiologies, such as hormonal disorders or hypoxia, with subsequent oxidative stress (OS) [3]. In women, this condition is caused mainly by polycystic ovary syndrome [4], chemotherapy, and radiotherapy due to cancer treatment [5]. Nevertheless, up to 25% of cases are idiopathic [6]. Several treatment possibilities exist for infertility, such as intrauterine insemination (IUI), in vitro fertilization (IVF), and intracytoplasmic sperm injection (ICSI) [7].

As mentioned above, reactive oxygen species (ROS) including superoxide (O^2•−^), hydrogen peroxide (H_2_O_2_), hydroxyl radical (OH^•^), and many others, may significantly contribute to infertility when their production exceeds the antioxidant defenses of reproductive cells [8]. ROS can affect cells negatively by damaging biomolecules, such as proteins, nucleic acid (NA), and lipids [9]. These damaged cells suffer from OS caused by an imbalance between ROS and antioxidants. OS could lead to cell death and the formation of pathologies, such as defective inflammatory response, oncogenesis [10], or disorders of organ systems [11]. Cells have developed various compounds, such as antioxidants, to protect themselves against ROS and OS.

Two main groups of antioxidants are non-enzymatic and enzymatic. The first group includes vitamin C [12], vitamin E, and antioxidants such as glutathione, thioredoxin, melatonin, carotenoids, and flavonoids. The second group includes more effective enzymes, such as superoxide dismutase, catalase, and glutathione peroxidase (GPx) [13].

The main function of GPx is to decrease hydro-peroxide (such as hydrogen peroxide or lipid hydroperoxide) levels [14] while water and corresponding alcohol are formed [15].

Overall, eight members (GPx1–8) of the GPx group are known in mammals. Their catalytic center consists of two main categories according to amino acid tetrad—SecGPx and CysGPx. Human GPx 1, 2, 3, 4, and 6 contain selenocysteine (Sec), and GPx 5, 7, and 8 contain cysteine (Cys) [16]. GPx 1, 2, 3, 5, and 6 belong to the homotetramers and GPx 4, 7, and 8 belong to the monomers [17].

GPx4 was detected in several cell organelles, including cytosol, mitochondria, nucleus, and endoplasmic reticulum (ER) [18]. This enzyme protects the cell against ROS formed in mitochondria using cytochrome C suppression and protects cell membranes from OS [19]. GPx4 plays a very important role in the vitality, motility, and morphological integrity of sperm cells containing three isoforms, nuclear/sperm nucleus-associated GPx4 (nGPx4/snGPx4) detected in the nucleus of sperm, cytosolic/cellular GPx4 (cGPx4) situated in the cytosol, and mitochondrial GPx4 (mGPx4) localized in mitochondria of the sperm middle piece. Here, mGPx4 probably acts as a structural protein without enzymatic activity. A study found that mGPx4 knockout mice were viable, but animals with a total GPx4 absence did not survive early embryogenesis [20], which means defective gastrulation [21]. In male mGPx4 knockout mice, the non-physiological organization of axoneme and impaired mitochondrial distribution in the sperm middle piece was observed, which resulted in oligoasthenozoospermia [20,22]. nGPx4 plays an important role in stabilizing condensed chromatin in testicles and the epididymis [23]. nGPx4 knockout animals showed no reproductive disorders, and they were fully viable. This phenomenon could be explained by the ability of cGPx4 to at least partially replace the absence of nGPx4 because cGPx4 can enter the sperm nucleus [24]. When performing a selective expression of only cGPx4, mice embryos survived [25]. Yant et al. (2003) asserted that cGPx4 is the only GPx4 isoform that is embryonically lethal in knockout animals [26]. Accordingly, the lack of cGPx4 could cause the defective development of mice embryos [22].

After implantation, decreased expression of GPx4 was detected in the human placenta, specifically in the microvillous membrane. This decrease was associated with pre-pregnancy obesity in women [27]. GPx4 also plays important role in preeclampsia in women, because decreased expression of this enzyme may lead to preeclampsia development [28]. On the other hand, the total lack of information about GPx4 involvement during the preimplantation period of pregnancy was the main stimulus for this research work.

## 2. Results

### 2.1. Immunofluorescent Analysis (IFA)

IFA was used to examine O/PE (oocytes/preimplantation embryos). O/PE were isolated, and the average number per female was calculated as the mean ± S.D. On D1, 52 O/PE, on D3, 53 PE, and on D5, 47 PE were flushed out of the oviduct/uterus, which means 10.4 ± 4.34 on D1, 10.6 ± 4.39 on D3, and 9.4 ± 3.78 on D5 O/PE per female. From the 52 O/PE isolated on D1, 30 were zygotes, which means a 58% fertilization rate.

Only the oocytes and zygotes on D1, the 2-cell and 4-cell embryos on D3, and the blastocysts on D5 were considered normal O/PE. Other stages were regarded as degenerated.

IFA for GPx4 detection was performed on half of the O/PE.

**Figure 1:**
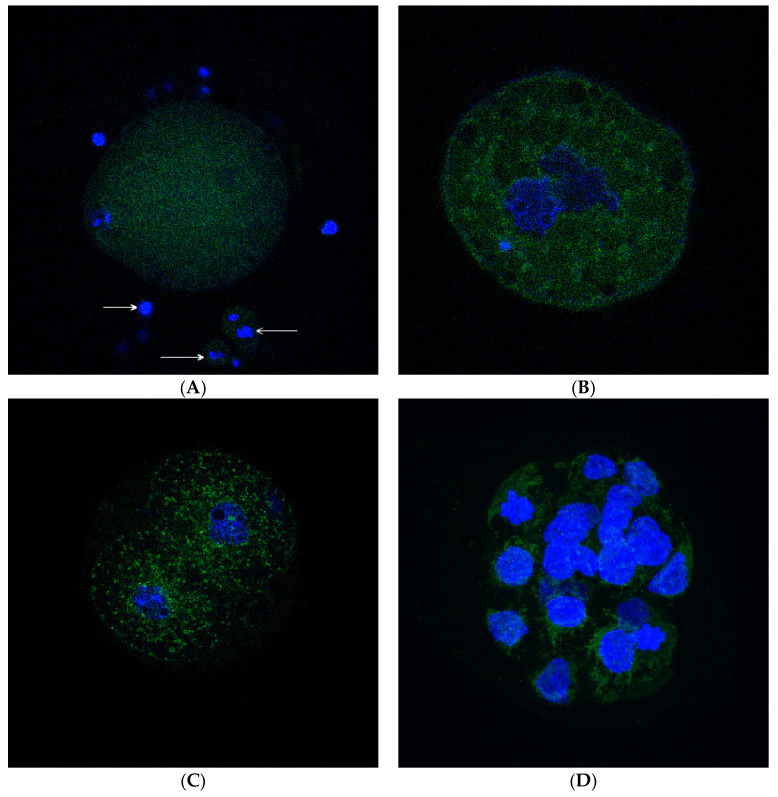

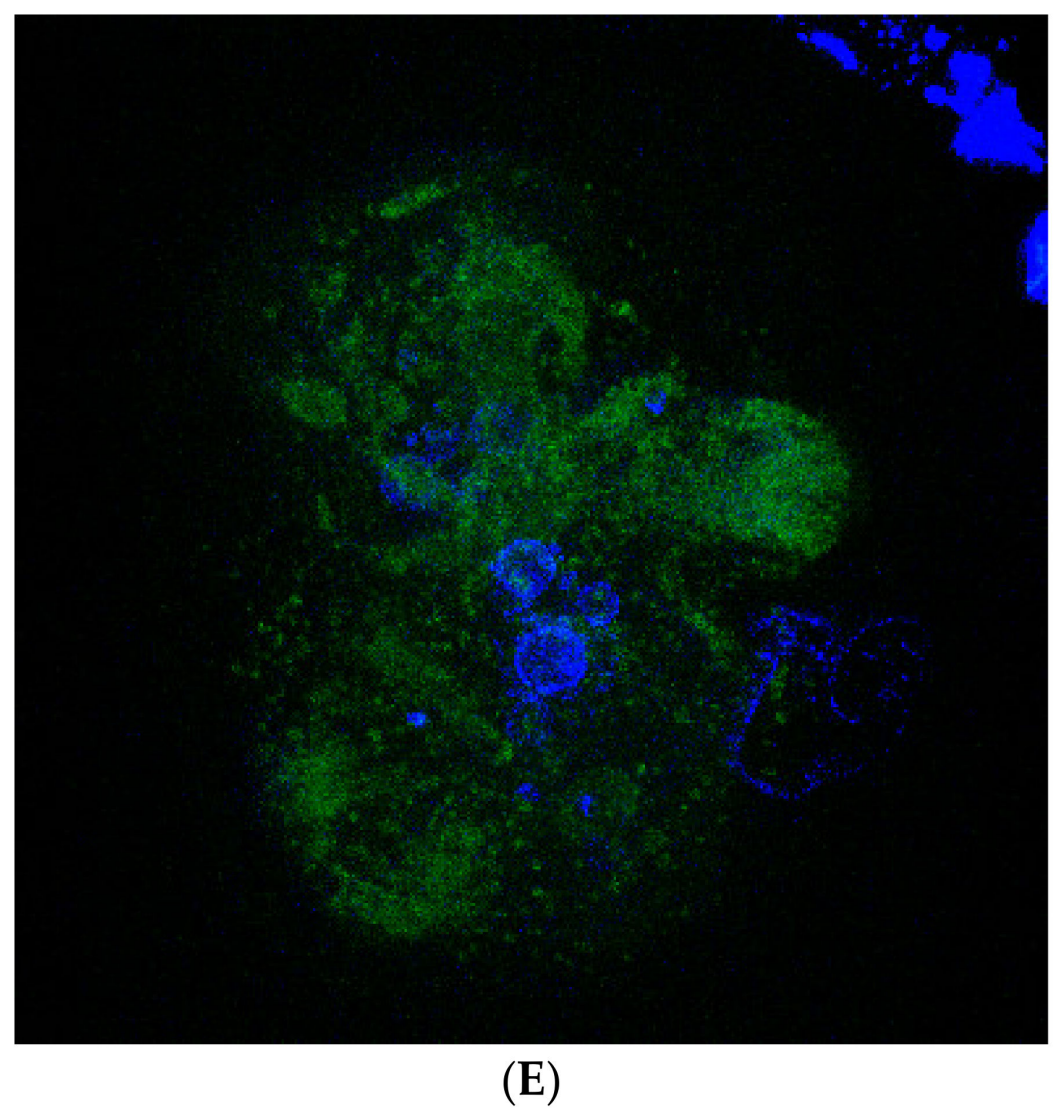
GPx4 presence in the rat oocyte and preimplantation embryos. (**A**) Shows the rat oocyte with cumular cells on D1 detected under a confocal microscope. The immunofluorescent analysis showed homogeneously distributed GPx4 in the cytoplasm of the oocyte and cumular cells (arrows). (**B**) Shows the rat zygote on D1. The immunofluorescent analysis shows the beginning of GPx4 cluster formation in the cytoplasm of the zygote on D1, detected under a confocal microscope. (**C**) Shows the rat 2-cell embryo on D3. The immunofluorescent analysis shows GPx4 clusters in the cytoplasm of the 2-cell embryo on D3, detected under a confocal microscope. Clusters are mostly in perinuclear space, less on the periphery. (**D**) Shows the rat blastocyst on D5. The immunofluorescent analysis shows GPx4 clusters in the cytoplasm of the blastocyst on D5, detected under a confocal microscope. Clusters are mostly in perinuclear space. (**E**) Shows the rat degenerated embryo. The immunofluorescent analysis shows a combination of homogeneous distribution and cluster formation of GPx4 detected under a confocal microscope. Blue represents DNA identified by Hoechst 33342; green shows GPx4 identified by an anti-GPx4 antibody conjugated with fluorescein isothiocyanate (FITC); GPx4 is glutathione peroxidase 4; D1, D3, and D5 refer to the corresponding days of pregnancy.

GPx4 was detected in all isolated O/PE stages. In the oocytes and granular cells (Figure 1A), the enzyme was distributed homogeneously in the cytoplasm. After fertilization, GPx4 formed many granules in the cytoplasm of the zygote (Figure 1B). A similar arrangement also persisted in 2-cell embryos (Figure 1C). At the blastocyst stage (Figure 1D), GPx4 was observed mostly around the nuclei rather than near the cell membrane. A combination of both homogeneous distribution and cluster formation was observed in degenerated embryos (Figure 1E).

### 2.2. Immunohistochemistry

**Figure 2:**
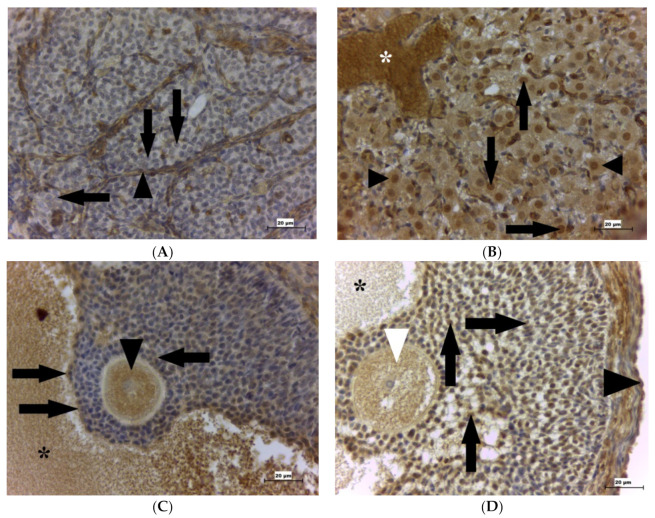
GPx4 presence in the rat ovary. (**A**) Shows the rat corpus luteum on D1. Immunohistochemical analysis shows no positivity of the nuclei and cytoplasm of the younger growing granulosa-lutein cells (arrows) for GPx4. Theca-lutein cells (arrowhead) are positive for the examined enzyme. (**B**) Shows rat corpus luteum on D1. Immunohistochemical analysis shows cytoplasmic (arrowheads) and nuclear (arrows) positivity of matured granulosa-lutein cells, and erythrocytes (white asterisk) for GPx4. (**C**) Shows the rat Graafian follicle with cumular cells on D1. Immunohistochemical analysis shows the positive cytoplasm of the oocyte (arrowhead) and cumular cells (arrows) and positive follicular fluid (asterisk) for GPx4. (**D**) Shows the rat atretic Graafian follicle with cumular cells on D5. Immunohistochemical analysis shows the positive cytoplasm of the oocyte (white arrowhead) and cumular cells (arrows), positive follicular fluid (asterisk), and germinal epithelium (black arrowhead) for GPx4. Scale bar = 20 μm. GPx4 is glutathione peroxidase 4; D1 is the first day of pregnancy; D5 is the fifth day of pregnancy.

GPx4 was detected in many cell types in the ovary. It was localized in the fibroblasts of perivascular space, the smooth muscle cells of the arterioles, the endothelial cells of the capillaries inside the corpus luteum, and blood plasma. Moreover, this enzyme was also found in the cells of the germinal epithelium, the cytoplasm and nuclei of the theca-lutein cells (Figure 2A) and granulosa-lutein cells of corpus luteum (Figure 2B), follicular cells at different stages of follicular development, cells of cumulus oophorus of the Graafian follicle (Figure 2C) and atretic Graafian follicle (Figure 2D), and the oocyte cytoplasm. GPx4 positivity was also detected in the follicular liquor.

**Figure 3:**
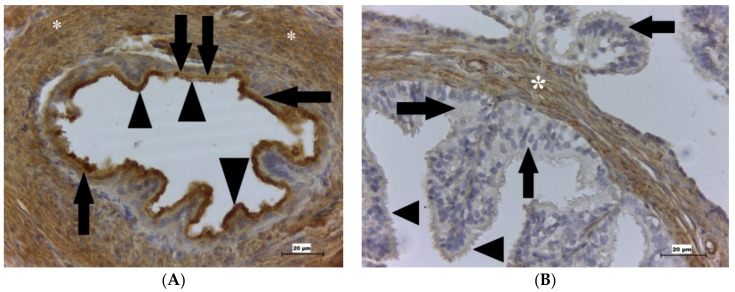
GPx4 presence in the rat oviduct. (**A**) Shows the rat oviduct on D1. Immunohistochemical analysis shows a cross-section of the oviduct. Kinocilia are strongly positive for GPx4 (arrowheads), the cytoplasm of the epithelial cells (arrows) and the smooth muscle cells (white asterisks) are also GPx4 positive. (**B**) Shows the rat oviduct on D5. Immunohistochemical analysis shows a cross-section of the oviduct. Kinocilia are GPx4 negative (arrowheads) together with the cytoplasm (arrows) of the epithelial cells, stromal cells (asterisk) are GPx4 positive. Scale bar = 20 μm. GPx4 is glutathione peroxidase 4; D1 is the first day of pregnancy; D5 is the fifth day of pregnancy.

GPx4 was found in the fibroblasts forming the stroma, the mesothelial cells, the endothelial cells and blood plasma, and the muscular layer. Furthermore, this enzyme was observed in the nuclei of the epithelial cells and also in their kinocilia on D1 and D3 (Figure 3A), but not on D5 (Figure 3B).

**Figure 4:**
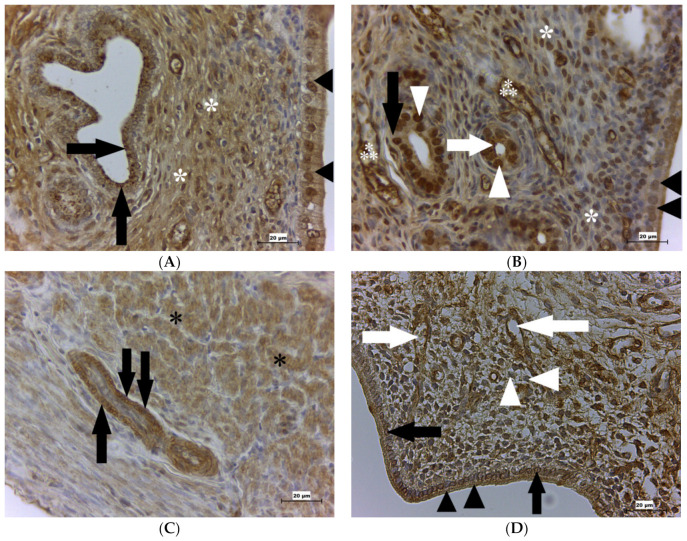
GPx4 presence in the rat uterus. (**A**) Shows the rat uterus on D1. Immunohistochemical analysis shows the GPx4 positivity of the endometrium, namely, granules inside the uterine glands (arrows) mostly in the apical part of the cells, stroma (asterisks), cytoplasm, and the nuclei (arrowheads) of the luminal epithelial cells. (**B**) Shows the rat uterus on D1. Immunohistochemical analysis of the endometrium shows GPx4 positivity of granules in the uterine gland epithelium (white arrow) mostly in the apical part of the cells, the nuclei (white arrowheads) and cytoplasm (black arrow) of the cells, in the blood vessels (⁂), stroma (asterisks), and luminal epithelial cells (black arrowheads). (**C**) Shows the rat uterus on D1. Immunohistochemical analysis shows the positivity of GPx4 in the smooth muscle cells in the myometrium (asterisks) and blood vessels (arrows). (**D**) Shows the rat uterus on D5. Immunohistochemical analysis of GPx4 shows the positivity of the cytoplasm (black arrowheads) and the nuclei (black arrows) of the epithelial cells, stellate-like fibroblasts (white arrowheads) of the endometrium, and blood vessels (white arrows). Scale bar = 20 μm. GPx4 is glutathione peroxidase 4; D1 is the first day of pregnancy; D5 is the fifth day of pregnancy.

On D1, GPx4 was localized abundantly in the epithelium of the uterine glands. In the apical part of these cells, this enzyme forms many prominent granules in the cytoplasm (Figure 4A). Diffused GPx4 positivity was also found in the nuclei (Figure 4B). GPx4 was further observed in the cytoplasm and nuclei in the epithelial cells lining the lumen of the uterus. This enzyme was additionally detected in the stromal cells (fibroblasts), endothelial cells, smooth muscle cells of the myometrium (Figure 4C), and mesothelial cells of the perimetrium.

On D3, GPx4 was found in the same types of cells as on D1, but the enzyme began to disappear from cells of the uterine glands. Moreover, almost no positivity was observed in the nuclei of the epithelial cells lining the lumen of the uterus.

On D5, the situation was similar to D3, but the enzyme almost completely disappeared from the cells of the uterine glands. GPx4 positivity started to be visible in the endometrial decidua formed by stellate-like fibroblasts (Figure 4D).

### 2.3. Western Blot Analysis

**Figure 5:**
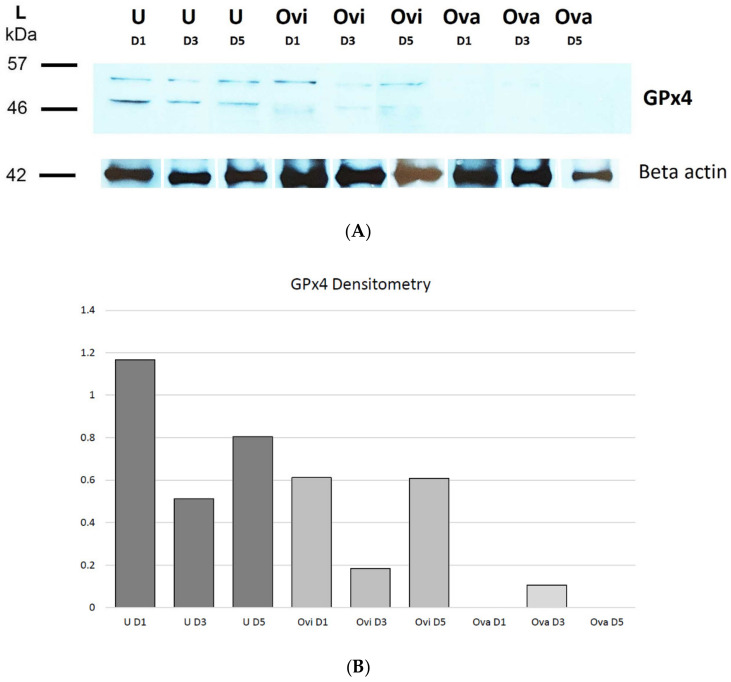
Western blot and densitometry analysis of the amount of GPx4 in rat female reproductive organs. (**A**) Shows Western blot analysis of GPx4. The amount of GPx4 in the rat reproductive organs compared to Beta-actin. 46 kDa could represent the mGPx4 dimer, and 57 kDa could be a coupling of mGPx4 together with nGPx4. (**B**) Shows densitometry of the female rat reproductive organs. Densitometry reveals the amounts of GPx4 in the rat reproductive organs compared to Beta-actin. Amounts of GPx4 were most abundant in the uterus on D1, but the enzyme on D1 and D5 was not detected in the ovary. U denotes uterus; Ovi is oviduct; Ova refers to the ovary; D1, D3, D5 denote the first, the third, the fifth day of pregnancy; GPx4 is glutathione peroxidase 4; mGPx4 refers to mitochondrial GPx4; nGPx4 is nuclear GPx4.

Samples of the uterus and oviduct showed two bands of different sizes (46 kDa and 57 kDa) on D1, D3, and D5, but the sample of the ovary showed only one very weak band (57 kDa) on D3 (Figure 5A).

Densitometry (Figure 5B) revealed that the amount of the GPx4 in the uterus was the most abundant on D1, decreased on D3, and slightly increased again on D5. An almost identical pattern was recorded for the GPx4 densitometry in the oviduct. In the ovary, the enzyme was not detected on D1 or D5, and on D3 its amount was even lower than the lowest amount of the enzyme in the oviduct or the uterus.

## 3. Discussion

In our work, we found that GPx4 was present throughout the entire preimplantation period of pregnancy from the unfertilized oocytes to the blastocysts. At the same time, we detected this enzyme in many cell types of the maternal ovary, oviduct, and uterus. The amount of the GPx4 enzyme varied between corresponding days of pregnancy in all examined organs.

Using IFA, we detected different GPx4 distribution in the cytoplasm of the oocytes and preimplantation embryos. This could be related to differences in the maternal and embryonic genome [29]. While the enzyme was distributed in the cytoplasm of the oocyte homogeneously, and GPx4 could play a structural role without enzymatic activity, after the fertilization, granules were formed mostly in the perinuclear region, which could be related to the ribosome and ER localization. In addition, it is also possible that GPx4, ranging from several hundred in one primordial germ cell [30] to approximately 100,000 in the fully developed oocyte in humans [31], is localized in mitochondria of the oocyte and embryo. However, after fertilization, a massive reduction of the mitochondrial amount occurs, and, in the blastocyst, only a small remnant of them remains in one cell [32]. Our results are supported by observing that in a 2-cell mouse embryo, many mitochondria accumulated around the nuclei [33]. This could be related to the metabolism of developing embryos, such as protein formation and mitochondrial respiratory processes. In the case of degenerated embryos, GPx4 not only forms granules but is also localized homogeneously in the cytoplasm. The reason for such an event could be ER and mitochondria destruction with the subsequent leak of GPx4 into the cytoplasm.

While lower ROS concentrations play important roles in ovulation, elevated levels could suppress many activities of the ovary [34]. GPx4 has an anti-apoptotic effect. It reduces phospholipid hydroperoxide and, thus, the cell damage processes [35]. Interestingly, Yoo et al. (2012) detected that adult mice with no GPx4 expression induced by tamoxifen died within 2 weeks after tamoxifen administration [36]. This indicates that GPx4 is an antioxidant enzyme that is vital for organisms and also for O/PE development. GPx4 is localized in several organs that have a direct or indirect relation to reproduction, such as reproductive organs (testis and oviduct), thyroid gland, and hypophysis [37]. Our result that GPx4 is present in rat ovary supports the finding of Yang et al. (2019) who reported the same finding [38]. GPx4 in this organ could play a role in the development of follicles because GPx4 can decrease levels of ROS and subsequently OS [39]. These authors also detected GPx4 in the corpus luteum and in both atretic and healthy bovine follicles, similar to our findings. Kankofer et al. (2013) found that the activity of GPx in the cow increased in the corpus luteum compared to the ovary [34]. This could be related to GPx1, which was detected in the granulosa cells of large follicles. These cells use elevated concentrations of cytochrome P450 to synthesize female reproductive hormones, such as estradiol-17β and progesterone. ROS are formed as byproducts during this synthesis and could lead to cell damage (such as oocyte) [40,41]. This could be related to other observations in our work. We detected that younger growing granulosa-lutein cells of the corpus luteum are negative for GPx4; however, matured granulosa-lutein cells with enlarged and GPx4 positive cytoplasm were observed, which could be related to steroid hormones synthesis, because enlarged granulosa-lutein cells are active and produce female reproductive hormones.

Cumulus oophorus cells are especially important for follicular viability and their further development. Follicular cells not only produce gonadal steroid hormones but also other substances, such as growth factors [42,43]. Furthermore, the granular cells influence the maturation of the oocytes through paracrine and junctional communication between the granular cells and the oocytes [44]. Apoptosis of granular cells leads to atresia of follicles [45,46].

GPx is present in the follicular fluid of both cows [47] and humans [48], suggesting that cumular cells could produce GPx4. Our findings that GPx4 is localized in cumular cells are consistent with the research of Fang et al. (2019), who observed GPx4 in the cumular cells of lambs [49]. We detected GPx4 not only in cumular cells and follicular fluid but also in the cytoplasm of the oocytes. Our results are also supported by the finding of GPx4 in bovine oocytes [50]. Positive follicular fluid could be related to the claim that precursors of selenium-dependent enzymes could originate from preovulatory oocytes because no selenium was detected in the uterine flushing from pigs [51]. Moreover, according to Ma et al. (2014), selenium in follicular liquor could originate from maternal blood, which in turn could affect the levels of selenium in this fluid [52]. It may suggest that the GPx4 observed in the follicular fluid could affect the correct course of the subsequent preimplantation processes. Selenium-dependent enzymes, such as GPx4, could be synthesized by 5-day porcine embryos [51]. Precursors of these enzymes could be derived from preovulatory oocytes. It has been reported that GPx4 in zebrafish was present in the cytoplasm of all blastomeres by the 512-cell stage [53]. This enzyme was also detected in the nuclei of zebrafish blastomeres.

On the other hand, in rats, we did not observe GPx4 in the nuclei of embryos, which could be related to the presence of selenium-dependent GPx4 in follicular fluid and in the cytoplasm of the rat oocytes and embryos. Our results extend knowledge obtained from mouse embryos where this enzyme was detected using the immunohistochemical method only from day D 8.5–9.5 of pregnancy [54]. Accordingly, an embryo might contain an antioxidant-enzyme pool, which could be represented as mRNA. This pool defends embryos against ROS [55].

Transcripts of GPx were found as mRNA in metaphase II oocytes in humans [56]. According to Meseguer et al. (2006), GPx4 presence in sperm cells can positively affect the development of the embryo, specifically its viability and symmetry [57]. GPx4 in the cytoplasm of O/PE may play a role in the preimplantation process because deficiency of cGPx4 situated in the cytosol of the cell could cause abnormalities and damage to the mice embryo caused by disorders of gastrulation. These embryos died in the uterus at the beginning of the second week of pregnancy (D7.5). Brutsch et al. (2015) also confirmed the importance of GPx4 because they detected an increased quantity of this enzyme in developing an embryo [54]. This indicates the importance of GPx4 in processes associated with maturation and fertilization [58] and embryo development [20,21,22]. Our data appears to fully support previous results [26] and even extend them to preimplantation embryos.

In the oviduct, we detected GPx4 in kinocilia, implying that the enzyme in this organ could have a similar function as in the sperm since mGPx4 acts as the structural protein in the middle piece, acrosome, and plasmatic membrane of the sperm. mGPx4 knockout mice showed abnormalities in sperm structure, such as unphysiological axoneme organization, separated sperm heads, and mitochondria distribution disorders of the middle piece. Sperm motility was also reduced, which led to oligoasthenozoospermia with subsequent male infertility [20,22]. If kinocilia are immobile, the oocyte does not move further into the uterine cavity but remains inside the oviductal lumen, leading to ectopic pregnancy.

Moreover, immobile kinocilia cannot form a proper barrier against defective sperm, resulting in the death of the embryo and unexplained infertility if the oocyte is fertilized by sperm with disorders. Furthermore, lower ROS levels have been found to stimulate the oviduct to maintain a suitable microenvironment that is important for the survival of sperm, oocytes, and embryos and for fertilization [59]. These authors further confirmed that estradiol increases GPx4 activity in female reproductive organs. Specifically, in the oviducts, the dominant follicle can affect GPx4 expression, since elevated enzyme concentration was detected during dominant follicle maturation and after its ovulation. GPx4 in the oviduct was found between the isthmus and the ampulla during the entire estrus cycle. We detected this enzyme by IHC in the epithelial cells and smooth muscle cells of the oviduct of rats, similar to Lapointe et al. (2005) who found that after the ovulation, the activity of GPx4 was the highest. This finding indicated that the ovary regulates GPx4 in the epithelium of the oviduct [59]. It can be suggested that estrogens support GPx4 formation, subsequently, protecting membranes of epithelial cells [19,59].

GPx activity was also detected in the endometrium and myometrium of the cow [34]. GPx formed by the uterus could improve the environment not only for a developing embryo but also for sperm, which passes through this organ. GPx activity was localized in the corpus luteum, uterine body, and horns [60]. Moreover, GPx activity was specifically found in the epithelial cells in the endometrial glands and stromal cells in humans [61]. In the glandular epithelium, GPx was observed in both apical and basal cytoplasm.

We also detected GPx4 in uterine glands, specifically in their nuclei and cytoplasm. In the cytoplasm, GPx4 forms granules, mainly in the apical part of the cells on D1. One can assume that these granules are secreted into the uterine cavity, where they could take part in improving the environment for sperm cells and preparing conditions for embryo implantation, as was described previously [60]. GPx4 was also found in the epithelial cells of the endometrium, which could correlate with the findings of the previously mentioned author. On the other hand, GPx4 in these cells could protect them against OS. We found GPx4 to be situated in fibroblasts in all examined organs. Our results confirm previous findings that GPx4 is situated in lung fibroblasts of mice [62]. In our study, we observed GPx4 in the decidua, specifically in stellate-like fibroblasts. Thus far, GPx4 has been detected in the uterus predominantly in the post-implantation period, namely in the amnion-chorion and villi of the human placenta [63], and in mice decidua on D 7.5 of pregnancy [64]. Our findings that GPx4 occurs in stellate-like fibroblasts suggest GPx4 presence in the uterus a few days earlier in the preimplantation period of pregnancy.

We detected GPx4 in the endothelial cells of blood vessels. This enzyme is important to secure proliferation together with redox homeostasis; otherwise, in its absence, ferroptosis occurs [65]. We found GPx4 in the endothelial cells of the ovary, oviduct, and also in the uterus and inside the erythrocytes in all examined organs. These findings support previous results, which suggested that GPx4 is vital for unsaturated fatty acids metabolism because their elevated levels can increase GPx4 levels and, subsequently, offer protection against inflammatory processes [66]. Moreover, GPx4 has been found in human endothelial cells of placental blood vessels by IHC [63].

In addition to the endothelial cells, we found GPx4 in the erythrocytes of all examined organs, which is consistent with previously published results [67]. Besides that, GPx4 is important for erythroid precursors in mice because the lower activity of this enzyme leads to the accumulation of toxic lipid intermediate products [68]. We detected GPx4 in the smooth muscle layer of blood vessels and smooth muscle cells in the oviduct and myometrium. GPx4 overexpression plays an important role in protecting aortic smooth muscle cells of rabbit against ROS, as was previously described [69].

Using WB, we detected GPx4 in all examined organs on almost all days of pregnancy. This confirms our previous results obtained using IHC. The only difference is that the ovary showed a very weak band only on D3, while on D1 and D5 the band was missing. This could be because the amount of GPx4 is too low to be detected by WB even if the enzyme is present in many cell types in the ovary. Methodological error is very unlikely because, with both methods (IHC and WB), we employed an antibody from one manufacturer. Except for the ovary, two bands of GPx4 were observed, which could be related to the presence of different isomers (cGPx4, mGPx4, nGPx4). Different sizes of bands could depend on post-transcriptional modification of the protein.

Since three different isoforms of GPx4 exist, their different molecular weights have also been described. The weight of nGPx4 is 34 kDa [23,70], the weight of cGPx4 is 20 kDa [71], and the weight of mGPx4 is 23 kDa [35]. It follows that the 46 kDa band in our work in the oviduct and uterus could represent the mGPx4 dimer, and the 57 kDa band found in all examined organs could be a coupling of mGPx4 and nGPx4. On the other hand, these different bands could represent cGPx4 after phosphorylation or glycosylation of protein, as this isoform is the most abundant and the only vital isoform of GPx4.

In our study, a pool of the organ samples on each day of pregnancy was used for WB detection. Because of that, we were not able to evaluate individual samples and perform statistical analysis, and further research is needed to clarify and supplement our results.

Concerning densitometry, on D1, the uterus showed the highest amount of this enzyme, which could be associated with sperm cells containing a certain amount of this enzyme. On D3, the amount of GPx4 was the lowest, probably due to the reduced amount of sperm. On D5, the levels of GPx4 increased possibly due to decidua formation [64].

The amount of GPx4 in the oviduct was the same on D1 and D5. The expression of GPx4 in cow was the highest after ovulation due to elevated concentrations of estradiol, which could be caused by the presence of sperm cells on D1. On D3, the GPx4 amount decreased, possibly due to the dropping of living sperm. On D5, the amount of enzyme was elevated. Compared to the ampulla, the muscular layer of the isthmus is thicker, considering the role of the isthmus in the contraction of the oviduct, moving embryos into the uterine cavity [59].

Surprisingly, we were not able to detect GPx4 in the ovary on D1 and D5 employing WB, but on D3, the amount of this enzyme increased. One can suppose that on D1, the corpus luteum is not so active in hormone production while on D3, the corpus luteum is more active and secrets progesterone, which is important to maintain pregnancy in the rat [72]. On D5, we expected the amount of GPx4 to be higher than on D3 because of corpus luteum development. Intensive metabolism in the corpus luteum during steroidogenesis probably also results in higher H_2_O_2_ production. Since catalase can degrade larger amounts of hydrogen peroxide than GPx [73], it might take over the function of GPx in the ovary. Therefore, GPx4 was not detected. However, why GPx4 was not presented on D5 requires further investigation.

## 4. Materials and Methods

### 4.1. Animals

The entire experiment involving animals was carried out according to the Committee for Ethical Control of Animal Experiments at Pavol Jozef Šafárik University (UPJŠ) and the State Veterinary and Food Administration of the Slovak Republic (permission no. Ro-11557/18-221/3, permission granted from 01/07/2018). A reduced number of animals was used in the experiment to minimize suffering. The Laboratory of Research Biomodels of the UPJŠ provided thirty Sprague Dawley rat females aged 85–90 days weighing approximately 320 g. Fifteen female rats were used for Western blot (WB), and the oocytes and preimplantation embryos were isolated from the oviduct and uterus. The remaining females (15 rats) were used for immunohistochemical detection of GPx4 in their reproductive organs. Standard diet and water were available to the animals ad libitum all the time. The daily cycle consisted of 12 h of light and 12 h of dark. Females were mated individually with males of the same strain from 7 a.m. to 9 a.m. Females were examined for the absence/presence of a vaginal plug. Its presence was a sign of successful mating. This day was marked as D1 (the first day of pregnancy). On the first, third, and fifth days of pregnancy (D1, D3, D5), five females in each group were overdosed by a lethal dose of Zoletil 100 mg/kg (Virbac SA, Carros, France).

### 4.2. Isolation of the Oocytes and Preimplantation Embryos

Oviducts and uterine horns from five female rats were removed on the corresponding day of pregnancy. O/PE were isolated under a stereomicroscope using a phosphate buffer solution (PBS) +BSA (3 mg/mL) as a rinse solution, similarly to Sefcikova et al., 2018 [74].

### 4.3. Immunofluorescent Analysis (IFA)

After isolation, O/PE were fixed for 1 h at room temperature (RT) in PBS containing 4% paraformaldehyde (PFA) (Merck, Darmstadt, Germany). Embryos were then stored at 4 °C in 1% PFA for a maximum of one week. O/PE were then transferred through three drops of PBS/BSA (0.1% BSA, referred to as the PBS/BSA next in the text) and permeabilization was performed. Cells were stored in PBS containing 0.5% Triton X-100 (Sigma-Aldrich, Saint-Louis, MO, USA) for 1 h at RT and were then washed in PBS/BSA two times for 5 min. Following overnight incubation at 4 °C in rabbit polyclonal anti-GPx4 antibody conjugated with FITC (#orb8194, 1:50, Biorbyt Ltd., Cambridge, UK), O/PE were washed six times for 5 min in PBS/BSA at RT. DNA was stained using Hoechst 33,342 (10 μL mL^−1^ in PBS; Sigma-Aldrich, Saint-Louis, MO, USA) for 5 min at RT. Embryos were again washed in PBS/BSA and mounted on the slides using Vectashield (Vector Laboratories, Burlingname, CA, USA). Finally, we examined and photographed O/PE under a confocal microscope (FV-1000 BX61; Olympus, Tokyo, Japan), as was described previously by Baran et al., 2013 [75].

### 4.4. Immunohistochemistry (IHC)

The ovaries, oviducts, and uterine horns were removed on D1, D3, or D5 and embedded in paraffin using the standard procedure. After that, paraffin blocks were cut into 5 μm thick sections. Slides were deparaffinized and rinsed using EnVision Flex Wash Buffer (#K800721-2, Agilent Dako, Santa Clara, CA, USA). Slides were incubated in a mixture of methanol and hydrogen peroxide to block endogenous peroxidase activity. EnVision Flex Wash Buffer (next referred to as wash buffer) was used for washing, followed by the revitalization of slides in the microwave. After another rinse with a wash buffer and 2% blocking solution of dried milk in Tris buffer, the rabbit primary anti-GPx4 polyclonal antibody (#bs-3884r, Bioss Antibodies Inc., Woburn, MA, USA) was added for overnight incubation at 4 °C, with a subsequent rinse in a wash buffer. The next step involved adding Biotinylated Link (#K0675, Agilent Dako, Santa Clara, CA, USA). Another wash was then performed, and Streptavidin-HRP (#K0675, Agilent Dako, Santa Clara, CA, USA) was added. After the next wash, 3,3-diaminobenzidine (DAB) (#K5207, Agilent Dako, Santa Clara, CA, USA) was used to visualize GPx4. Tap water was employed for washing, and slides were stained with hematoxylin and mounted into Pertex, similarly to Liu et al., 2020 [76].

To obtain negative controls, the entire procedure was repeated omitting the primary antibody. Slides were evaluated under a light microscope independently by two observers. GPx4 positivity or negativity of the different cell types, extracellular matrix, blood plasma, and follicular fluid was observed on each day of pregnancy (D1, D3, D5). Five serial sections from all animals were obtained to be sure that all layers and all possible cell types present in the organs were found for evaluation. Sections were evaluated and compared with each other to detect if sections had the same pattern.

### 4.5. Western Blot (WB)

After isolating O/PE, ovaries, oviducts, and uterine horns were stored in Eppendorf tubes at −80 °C. Organs were washed two times using ice-cold PBS. RIPA buffer (1x PBS, 1% Nonidet P-40, 0.5% sodium deoxycholate, 0.1% sodium dodecyl sulfate-SDS), and protease and phosphatase inhibitor cocktail (all chemicals bought from Thermo Fisher Scientific, Inc., Waltham, MA, USA) were used for homogenization. Obtained lysates were incubated on ice for 45 min, and sonication was performed for 30 s at 30 V (Sonopuls HD 2070; Bandelin electronic GmbH & Co. KG, Berlin, Germany). Centrifugation (10,000× *g*) was performed at 4 °C for 10 min, and the supernatant was obtained and put into a microcentrifuge tube. After the separation of samples using 12% SDS-polyacrylamide gel, electroblotting was performed using Immobilon-P transfer membrane (Millipore Co., Billerica, MA, USA). Primary antibodies anti-GPx4 (#bs-3884r, Bioss Antibodies Inc., Woburn, MA, USA) and anti-β-actin (clone AC-74, 1:10,000; Sigma-Aldrich, Saint-Louis, MO, USA) were used for detection. Membranes with secondary horseradish peroxidase-conjugated antibodies (goat anti-rabbit IgG F (AB’) 2, 1:10,000, PI-31461 and goat anti-mouse IgG F (AB’) 2, 1:10,000, PI-31436, Pierce, Rockford, IL, USA) were incubated for one hour. ECL Western blotting substrate (PI-32106, Pierce, Rockford, IL, USA) and Kodak Biomax film (#1788207, Sigma-Aldrich, Saint-Louis, MO, USA) were used to visualize antibody reactivity. After visualization, GS-800 Calibrated Densitometer was applied to scan the films. Image J software version 1.52 (NIH, National Institute of Health, Bethesda, MD, USA) was employed to quantify protein, as was described previously by Feckova et al., 2019 [77].

## 5. Conclusions

To our knowledge, this is the first paper describing GPx4 expression in ovulated oocytes and preimplantation embryos in mammals. The enzyme was present in all developmental stages of a new individual, from the unfertilized oocytes to the blastocysts, which testifies its high importance in the reproductive process. On the other hand, we detected GPx4 in many specialized cells in all three examined female genital organs of the mother. It should be mentioned that in some specific structures, such as kinocilia, the enzyme acts as the structural protein without enzymatic activity, as has been previously described in sperm. Moreover, we observed nGPx4 in granulosa-lutein cells of the ovary, oviductal and uterine epithelium, and the cells of uterine glands even though nGPx4 is considered to be a specific enzyme in sperm nuclei. This could mean that nGPx4 is not as rare as expected and is not limited to sperm. On the other hand, cGPx4 migrated into the nuclei of these cells, as was previously described in males. Interestingly, the amount of GPx4 in the oviduct and uterus was the lowest on the day (D3) when the amount of GPx4 in the ovary was the highest. For now, we do not have a complete explanation for this phenomenon. Other studies are needed to clarify these processes. Based on our results, GPx4 is probably already important during the preimplantation period of pregnancy.

## Data Availability

All data are available at The Department of Anatomy, Medical Faculty, Šafárik University, Šrobárova 2, 041 83 Košice, Slovakia, Andrea Kreheľová, andrea.krehelova@student.upjs.sk.

## Abbreviations

BSA	Bovine serum albumin
cGPx4	Cytosolic/cellular glutathione peroxidase 4
Cys	Cysteine
D1–D5	Corresponding day of pregnancy
FITC	Fluorescein-5-isothiocyanate
GPx	Glutathione peroxidase
GPx4	Glutathione peroxidase 4
H_2_O_2_	Hydrogen peroxide
IFA	Immunofluorescent analysis
IHC	Immunohistochemistry
IgG	Immunoglobulin G
mGPx4	Mitochondrial glutathione peroxidase 4
nGPx4	Nuclear/sperm nucleus-associated glutathione peroxidase 4
O/PE	Oocytes/preimplantation embryos
OS	Oxidative stress
PBS	Phosphate buffer saline
ROS	Reactive oxygen species
RT	Room temperature
Sec	Selenocysteine
WB	Western blot
